# Dexmedetomidine improves acute lung injury by activating autophagy in a rat hemorrhagic shock and resuscitation model

**DOI:** 10.1038/s41598-023-31483-1

**Published:** 2023-03-16

**Authors:** Yifu Lu, Hiroko Shimizu, Ryu Nakamura, Yaqiang Li, Risa Sakamoto, Emiko Omori, Toru Takahashi, Hiroshi Morimatsu

**Affiliations:** 1 Department of Anesthesiology and Resuscitology, Okayama University, Graduate School of Medicine, Dentistry, Pharmaceutical Sciences, 2-5-1, Shikatacho, Kita-ku, Okayama-shi, Okayama 700-8558 Japan; 2 Department of Anesthesiology and Resuscitology, Okayama University Medical School, 2-5-1, Shikatacho, Kita-ku, Okayama-shi, Okayama 700-8558 Japan; 3 Okayama Saidaiji Hospital, 1-1-70 Kanaokahigashi machi, Higashi-ku, Okayama-shi, Okayama 704-8194 Japan

**Keywords:** Experimental models of disease, Macroautophagy

## Abstract

Dexmedetomidine (DEX) can reduce lung injury in a hemorrhagic shock (HS) resuscitation (HSR) model in rats by inhibiting inflammation. Here, we aimed to investigate if these effects of DEX are due to autophagy activation. Therefore, we established HSR rat models and divided them into four groups. HS was induced using a blood draw. The rats were then resuscitated by reinjecting the drawn blood and saline. The rats were sacrificed 24 h after resuscitation. Lung tissues were harvested for histopathological examination, determination of wet/dry lung weight ratio, and detection of the levels of autophagy-related marker proteins LC3, P62, Beclin-1, and the ATG12-ATG5 conjugate. The morphological findings of hematoxylin and eosin staining in lung tissues and the pulmonary wet/dry weight ratio showed that lung injury improved in HSR + DEX rats. However, chloroquine (CQ), an autophagy inhibitor, abolished this effect. Detecting the concentration of autophagy-related proteins showed that DEX administration increased LC3, ATG12-ATG5, and Beclin-1 expression and decreased P62 expression. The expression levels of these proteins were similar to those in the HSR group after CQ + DEX administration. In summary, DEX induced autophagic activation in an HSR model. These findings suggest that DEX administration partially ameliorates HSR-induced lung injury via autophagic activation.

## Introduction

Hemorrhagic shock (HS) is a type of hypovolemic shock. Perioperative hemorrhage, maternal hemorrhage, gastrointestinal hemorrhage, and aneurysm rupture are the main causes of HS^1^. Severe HS leads to pulmonary inflammation and acute lung injury^2–5^. Inflammation-derived serious lung injury is named as acute respiratory distress syndrome, and its prognosis after hemorrhagic shock resuscitation (HSR) is poor^6,7^. Therefore, developing effective therapeutic strategies to prevent acute lung injury during resuscitation is important.

Dexmedetomidine (DEX) is an alpha 2-adrenergic receptor agonist with a wide range of pharmacological properties. DEX can reduce sedative and opioid dose, stabilize hemodynamics, exert anti-inflammatory effects, and protect organs, among other pharmacological properties and has a wide range of clinical applications^8^. In recent years, several studies have confirmed its potential anti-inflammatory effects in various animal models. In different animal and cytological models, DEX can affect different signaling pathways and their downstream molecules, such as Toll-like receptor 4 (TLR4)/ primary response gene 88 (MyD88)/nuclear factor-κB (NF-κB), thereby inhibiting the release of pro-inflammatory factors and playing an anti-inflammatory role^9–12^. Previous studies have shown that DEX can effectively reduce systemic inflammation induced by HS, exerting a direct protective effect against HS-induced acute lung injury^13^.

Phagocytosis of unwanted cytoplasmic contents, followed by fusion and degradation with lysosomes is collectively known as autophagy^14,15^. Current studies have shown that autophagy exerts an anti-inflammatory effect on phagocytosis and cytoplasm degradation in all responses that can activate cell-autonomous inflammation^16^. As both DEX and autophagy have anti-inflammatory properties, it is unclear whether the protective effect of DEX on acute lung injury is related to autophagy. Therefore, exploring this potential connection may be helpful for understanding the protective mechanisms of DEX in acute lung injury.

This study aimed to investigate the effects of DEX on HSR-induced lung injury and autophagy in rats. We hypothesized that DEX exerts anti-inflammatory effects by activating autophagy. Additionally, we used chloroquine (CQ) as an autophagy inhibitor to verify the effect of DEX on autophagy.

## Results

### Effect of DEX on HSR-induced lung histological injury

**Figure 1:**
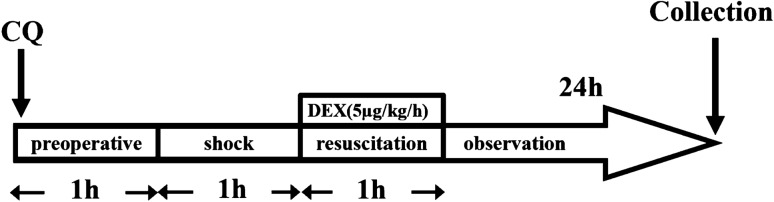
Establishment of the hemorrhagic shock resuscitation (HSR) rat model and chloroquine (CQ) and dexmedetomidine (DEX) administration schedule. The experimental animals were divided into six groups (n = 5–12 per group). Sham group: rats without hemorrhagic shock, normal saline (NS) was used instead of DEX and CQ; CQ + Sham group: Sham rats were pretreated with CQ 1 h in advance; NS + HSR + NS group: established rat HSR model using NS to replace DEX and CQ; NS + HSR + DEX group: HSR rats treated with DEX, and NS used instead of CQ; CQ + HSR + DEX group: CQ was intraperitoneally injected before the operation, and DEX was administered to HSR rats; CQ + HSR + NS group: HSR rats were pretreated with CQ 1 h in advance.

**Figure 2:**
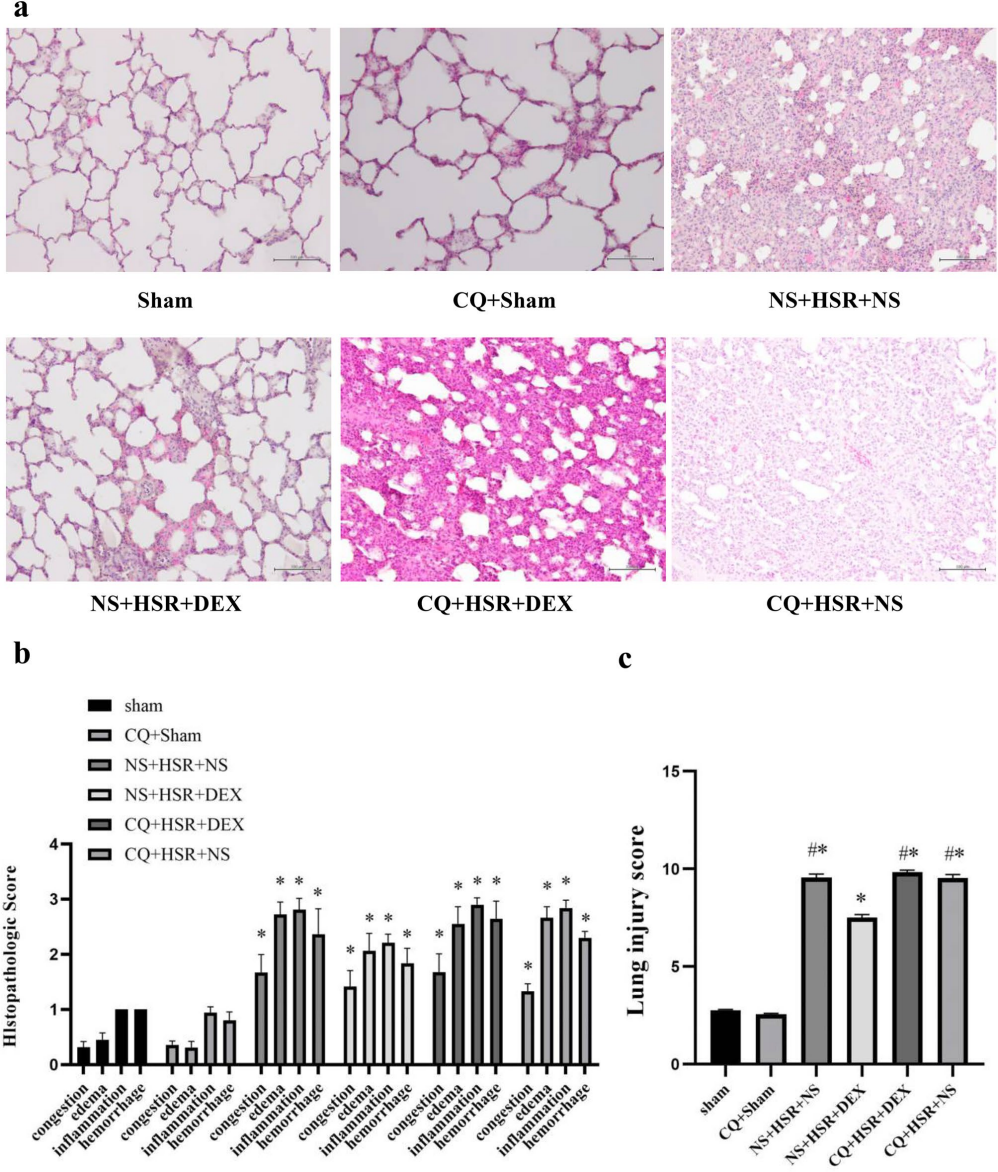
Histological examination of lung injury after hemorrhagic shock resuscitation (HSR). The cells were observed under a microscope (original magnification × 400). (**a**) Histological damage, such as congestion, edema, inflammation, and hemorrhage, was observed in the normal saline (NS) + HSR + NS group. However, these histopathological changes were markedly improved by dexmedetomidine (DEX) treatment. (**b,c**) The severity of lung histopathological changes was evaluated and scored based on four aspects: congestion, edema, inflammation, and bleeding. 0, none or normal; 1, mild; 2, moderate; and 3, severe. The pathological scores of the four parameters were calculated, and their sum in each group was used as the score for lung tissue injury. Consistent with the histological findings, the lung injury scores of the NS + HSR + NS group were significantly higher than those of the sham group; however, DEX treatment significantly reduced the score, and CQ had no significant effect on the score. Statistical analysis was performed using ANOVA followed by the Tukey–Kramer multiple-comparison test between groups. *p < 0.05 (vs. sham) #*p* < 0.05 (vs. NS + HSR + DEX).

We assessed the effect of DEX on HSR-induced lung injury 24 h after resuscitation in rats via microscopic observation of hematoxylin and eosin-stained lung sections at 400X magnification. Histological damage, such as congestion, edema, inflammation, and hemorrhage, was observed in the normal saline (NS) + HSR + NS group. DEX treatment markedly ameliorated these histopathological changes (Fig. 2a). Consistent with the histological findings, the lung injury scores of the NS + HSR + NS group were significantly higher than those of the sham group; however, DEX treatment significantly reduced this score (Fig. 2b,c). As Fig. 1 shown that pretreated with the autophagy inhibitor CQ, the effects of DEX on lung histopathology were attenuated in rat lung tissues, while CQ alone had no significant effect on the lung histopathology of rats. The lung injury scores of the CQ + Sham and CQ + HSR + NS groups were not significantly different from those of the Sham and NS + HSR + NS groups, but the results of various lung injury histological examination scores of the CQ + HSR + DEX group were higher than those of the DEX-treated group without autophagy inhibitors (Fig. 2b). Importantly, the lung-protective effect of DEX was inhibited, and the lung injury score increased significantly in the CQ + HSR + DEX group, and the scores were almost the same as those of the NS + HSR + NS group (Fig. 2c). These results suggested that autophagy plays a crucial role in the protective effects of DEX against HSR-induced lung injury.

### Effect of DEX on lung wet/dry weight ratio in HSR model rats

**Figure 3:**
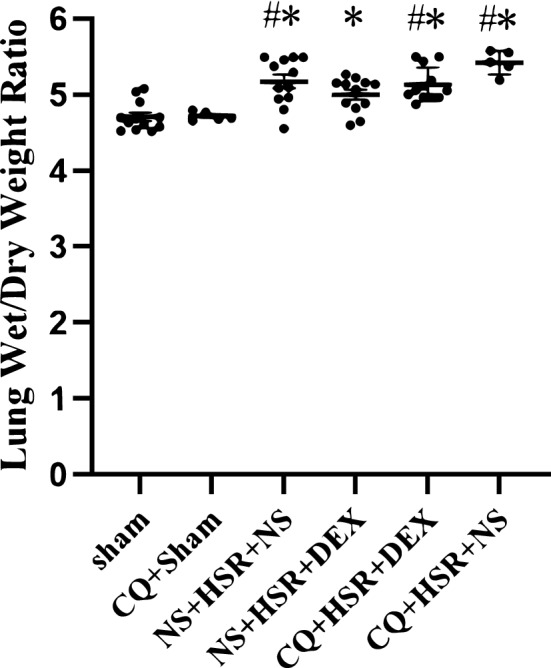
Effect of dexmedetomidine (DEX) administration on lung edema after hemorrhagic shock resuscitation (HSR). Effects of DEX on HSR-induced lung wet/dry weight ratio. Lung wet/dry weight ratio. Twenty-four hours after HSR, the left lung of each rat was excised and weighed (wet weight), placed in a 110 °C environment to dry for 24 h, and then weighed again (dry weight). The lung wet/dry weight ratio was calculated by dividing the wet weight of the lung by the dry weight to evaluate pulmonary edema in HSR rats. Statistical analysis was performed using ANOVA followed by the Tukey–Kramer multiple-comparison test between groups. *p < 0.05 (vs. sham) #*p* < 0.05 (vs. NS + HSR + DEX).

To determine the effect of DEX on lung injury, we assessed the lung wet/dry weight ratio as an index of pulmonary edema in the different groups of rats. The lung wet/dry weight ratio 24 h after resuscitation of the NS + HSR + NS group was higher than that of the sham group (Fig. 3). However, DEX treatment following HSR significantly decreased HSR-induced lung edema (Fig. 3). In addition, we found that CQ + Sham group and CQ + HSR + NS group showed that the use of CQ alone in the model did not significantly aggravate pulmonary water, the difference was not statistically significant compared with the Sham group and NS + HSR + NS group, but before DEX intervention, the degree of pulmonary edema in the CQ + HSR + DEX group, where autophagy inhibitors were used before surgery, was more severe than that in the DEX treatment group (Fig. 3). This result is consistent with that of the histological examination of the lungs of HSR rats, suggesting that DEX improved HSR-induced pulmonary edema, whereas CQ inhibited this effect.

### Effects of DEX on autophagy markers

**Figure 4:**
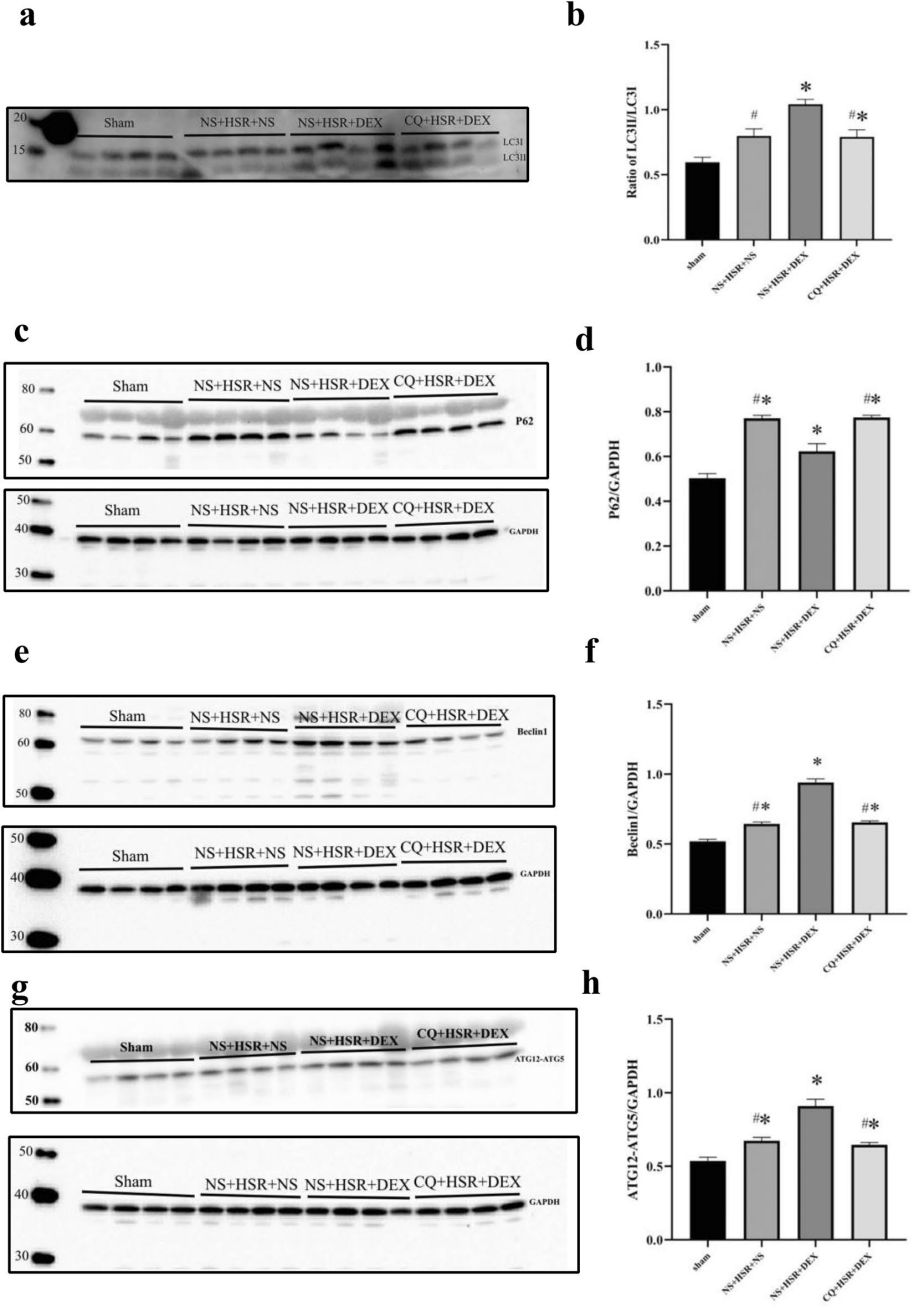
Effect of dexmedetomidine (DEX) injection on autophagy marker proteins in the lungs following hemorrhagic shock resuscitation (HSR). The right lung tissue of HSR rats was resected 24 h after resuscitation, protein extraction was performed, and the LC3, P62, Beclin-1, and ATG12-ATG5 expression levels were measured via western blotting. (**a**,**c**,**e**,**g**) Representative LC3, P62, Beclin-1, and ATG12-ATG5 immune responses in the four rat lung tissue groups with different treatment blots. GAPDH served as a loading control. (**b**,**d**,**f**,**h**) In western blot analysis, the LC3II grayscale measurement was normalized to the expression level of LC3I, and GAPDH expression level was used as the grayscale measurement standard for P62, Beclin-1, and ATG12-ATG5. Protein expression levels were quantified using Image Lab Version 5.0. Data are presented as mean ± standard error (n = 12 per group). Statistical analysis was performed using ANOVA, followed by the Tukey–Kramer multiple-comparison test between groups. *p < 0.05 (vs. sham) #*p* < 0.05 (vs. NS + HSR + DEX).

We performed western blotting to detect the levels of autophagy marker proteins, such as LC3, P62, Beclin-1, and the ATG12-ATG5 conjugate. Compared with the control group, LC3II/LC3I level increased significantly in the NS + HSR + DEX group, while that of LC3II/LC3I decreased after CQ pretreatment to levels lower than those in the DEX treatment group, and almost the same as those in the HSR group (Fig. 4a,b). We also found that the P62 protein expression level decreased significantly in the DEX treatment group. P62 expression levels in the HSR and CQ pretreatment groups were similar, and both were higher than that in the DEX treatment group (Fig. 4c,d). In addition, as shown in Fig. 4e,f, Beclin-1 protein expression in the NS + HSR + DEX group was significantly higher than that in the other groups, and it decreased in the CQ + HSR + DEX group owing to the influence of autophagy inhibitors. Finally, ATG12-ATG5 conjugate expression increased significantly in the NS + HSR + DEX group, and it was significantly higher than that of the other groups (Fig. 4g,h). Similar to the LC3 and Beclin-1 assays, ATG12-ATG5 conjugate expression was also affected by CQ in the CQ + HSR + DEX group and was similar to that in the NS + HSR + NS group. These results show that the protective effect of DEX on the lungs correlated positively with autophagy.

## Discussion

This study showed that DEX treatment before HSR can effectively alleviate HSR-induced acute lung injury, and pre-administration of CQ did not cause additional effects on lung tissue, but when used with DEX, abolished the protective effects of DEX. This was demonstrated via lung tissue histological staining to assess lung injury and the lung wet/dry weight ratio to assess pulmonary edema. In addition, we found that DEX affected autophagy in the lungs of the rat HSR model, as indicated by the changes in the expression of the autophagy-related proteins LC3, P62, Beclin-1, and ATG12-ATG5. These changes were abolished by the autophagy inhibitor CQ. Therefore, DEX acts as an adjuvant therapy to reduce lung injury after HSR, at least partially by regulating autophagy.

In our study, tissue staining results revealed that lung tissue damage improved significantly in the DEX treatment group. However, the effect of DEX on the lung tissue was abolished in the CQ group. In previous studies, DEX has also shown protective effects against lung damage. A single large dose of DEX administered 24 h before surgery could reportedly activate autophagy to prevent myocardial dysfunction in septic rats^17^. In our study, we investigated the effect of continuous DEX administration on autophagy during the resuscitation phase and its relationship with HSR-induced lung injury. As one of the most widely used autophagy inhibitors, CQ can block the fusion of autophagosomes and lysosomes in the final stage of autophagy^18^. Currently, no studies have shown that the use of CQ can aggravate lung injury. In this study, we found that the use of CQ alone did not cause lung injury, and the use of CQ did not significantly aggravate lung injury in the HSR model. Therefore, CQ successfully weakened the protective effect of DEX on HSR-induced lung injury in the autophagy inhibition experiment and the CQ itself had no effect on HSR lung injury. This result verified that DEX exerts a protective effect on HSR-induced lung injury, which CQ can reverse, indicating that it is related to autophagy.

In the present study, we found that DEX increased the expression of autophagy-related proteins LC3, Beclin-1, and ATG12-ATG5 and decreased that of P62 protein in the lung tissues of HSR rats. However, CQ administration attenuated the effects of DEX on autophagy-related proteins. LC3 western blotting is an effective mean to monitor autophagy, as the transformation of LC3I to LC3II marks autophagy activity^19^. Studies have shown that P62 accumulates, and its expression increases if the degradation of ubiquitinated proteins by autophagosomes and lysosomes is blocked, whereas it decreases otherwise^20^. Studies have shown that Beclin-1 can activate autophagy and is positively correlated with the expression of the autophagy-specific marker LC3^21^. ATG5 is one of the most commonly targeted proteins in autophagy protein analyses. It commonly binds to ATG12, and the complex is positively correlated with LC3 and Beclin-1 and negatively correlated with P62 during autophagy^22^. Therefore, changes in autophagy can be observed by detecting the levels of these four proteins. Here, DEX enhanced autophagic activity in the lung tissues of HSR rats, which was inhibited by CQ.

During HSR, progressing from hypoxia to reoxygenation and neutrophil activation both produces large amounts of reactive oxygen species (ROS), which enhances the systemic inflammatory response and ultimately leads to lung and other multi-tissue damage^23,24^. Recent studies have shown that phosphorylation of AMP-activated protein kinase (AMPK) by autophagy-activating kinase 1 is required for autophagy^25^. Furthermore, other studies have shown that DEX increases AMPK expression and reduces ROS production in a mouse ischemia/reperfusion model^26^. Our results suggested that autophagy plays a positive role in the protective effects of DEX, which can effectively alleviate lung injury. Therefore, whether DEX enhances autophagy via the AMPK pathway to attenuate HSR-induced lung injury requires further investigation.

At present, there were studies through other models such as the sepsis model and the toxic shock model, confirmed that DEX plays an organ protective role through autophagy^27,28^. However, the inductions for establishing the models are different, and the mechanism of action also varies with the pathology. Meanwhile, Shankun Zhao et al. also found that some studies showed that DEX treated organ injury by inhibiting autophagy, while other studies showed that DEX significantly increased autophagy levels in the process of protecting against organ injury^29^. In addition, autophagy has been reported to aggravate lung injury, and the use of autophagy inhibitors reduces the inflammatory response in lung tissue^30^. Therefore, we speculate that this may be related to over-activation of autophagy, whose level and role also change at different stages of lung injury, and the effect of DEX on autophagy in the process of organ protection also changes accordingly^31^. To the best of our knowledge, this study is the first to demonstrate that DEX exerts the lung protective role by activating autophagy in the HSR model. Although regrettably, we have not yet elucidated its mechanism of action, we believe that in clinical practice these results can support the selection of DEX in patients who are resuscitated from hemorrhagic shock. The mechanism of DEX affecting autophagy and the relationship between autophagy and organ injury will be our future research direction.

It is worth noting that autophagy is a dynamic, multi-step, and complex degradation process. This study only examined the effect of DEX on lung injury after 24 h in the rat HSR model; moreover, our choice of the autophagy inhibitor CQ only affects the downstream autophagy flow. In this case, we can only examine the effect of DEX on autophagy, but cannot explain its effect on the entire autophagy flow. Furthermore, western blotting can only measure the average autophagy protein levels and cannot show the complete development of autophagy in cells^20^. Therefore, more accurate methods are needed to detect autophagy at different time points in subsequent studies to obtain more comprehensive data on the autophagic flow. Additionally, the proteins examined in this study play a crucial role in typical autophagy, but studies have shown that LC3, Beclin-1, and ATG5-ATG12 are also expressed in other non-classical autophagy processes, even independent autophagy^32^. Therefore, the impact of DEX on the non-canonical autophagy pathways involving these proteins still needs to be studied. Finally, although studies have confirmed that DEX, an α2-adrenergic receptor agonist, can enhance autophagy through the AMPK signaling pathway^33^, no other α2-adrenergic receptor agonists have been found to activate autophagy. Therefore, it is inferred that the effect of the α2-adrenergic receptor on autophagy is a necessary, but insufficient factor. There are other factors that cause DEX to affect autophagy, which should be explored further.

In addition, this study has several limitations. First, we did not use Berlin definition to define our ALI^34^. Actually, we did not use mechanical ventilation at all in this study. In the previous study, we established the HSR model and judged the lung injury in the model through data such as PaO_2_, but there was no significant difference in PaO_2_ in the sham group and HSR group^35^. Instead of PaO_2_, it has been reported that histological examination of lung tissue damage is one of the most direct pieces of evidence for identifying ALI, and ALI also can be further validated by measuring the changes in the alveolar-capillary barrier through the lung wet/dry weight ratio^36^. Therefore, in this study, PaO_2_/F_I_O_2_ ratio and ventilator settings were not used to judge lung injury, but the lung tissue wet/dry weight ratio and HE staining in the HSR model were used to evaluate lung injury. Second, we did not show survival rate in this study, because this study aims to observe the effect of DEX on autophagy, but not survival rate. So all rats survived after the successful establishment of the HSR model. Therefore, we could not get the relevant data on survival rate in this study setting. We believe our model is not lethal, but severe enough to assess lung injury after HRS. In conclusion, the results of this study suggest that the use of DEX before HSR can ameliorate HSR-induced lung injury, which is related to autophagy activation in the lungs. However, further studies are required to elucidate the effect of DEX on autophagy flux, as well as its mechanism of action.

## Methods

### Animals

This study was approved by the Animal Use and Care Committee of the Okayama University Medical School (OKU-2020813 on February 22, 2021, and OKU-2021014 on April 1, 2021) and conformed to the Guidelines for the Care and Use of Laboratory Animals based on the ARRIVE^37^ and 2020 AVMA euthanasia guidelines^38^. Male Sprague–Dawley rats (Clea Japan, Inc., Tokyo, Japan) weighing 350–450 g were housed in cages in 25 °C temperature-controlled chambers under a 12 h light/dark cycle (lights on at 08:00 h) with free access to food and water. A total of 58 rats were used in this study.

### HSR protocol

Under isoflurane anesthesia inhalation, a catheter was inserted into the tail vein for DEX administration, and the HSR model was created as previously described^39–41^. First, the left femoral artery and vein were dissected aseptically, and a heparinized polyethylene catheter was implanted. Arterial blood pressure monitoring and blood extraction were performed from the left femoral vein through the catheter during HSR model construction. Blood was collected from the left femoral vein into a heparinization syringe (10 units/ml) within 15 min, and rat mean arterial blood pressure was maintained at 30 mmHg for 45 min. Blood transfusion was performed for 15 min to recover the rats, and their vital signs were observed for 45 min. The surgical incision was then closed. Rats in the sham operation group underwent all surgical procedures, except for blood extraction. Throughout the experiment, the rats were able to breathe spontaneously without endotracheal intubation. All of the above operations were performed on heating pads to regulate the rectal temperature in rats within the physiological range.

All rats were grouped and marked in order of purchase and placed in cages of the same size. Each experiment was conducted in the same place in the order described above. Healthy viable rats were used in the experiments. Rats that died unexpectedly or were extremely weak were excluded from the study. In this study, there were four rats died unexpectedly during the resuscitation period of the making model. In addition, during the observation period after the model was successfully established, we assessed whether the rat was in intolerable pain by weight loss of ≥ 20% or a substantial decline in mobility, while defining this as the humane endpoint of the rat and immediately stopped the experiment and euthanized the rat an overdose isoflurane inhalation. This was done to minimize pain in the rats at any point in the experiment. However, no rats were euthanized due to this condition during the observation period of this study.

### Drug preparation

DEX (Maruishi Pharmaceutical Co., Ltd. Osaka, JAPAN) was dissolved in NS and adjusted to an adequate dose (5 µg/kg body weight in 2 ml saline) prior to injection. CQ diphosphate salt (Sigma-Aldrich Co., St. Louis, MO, USA) was dissolved in NS to obtain a 2 mg/ml stock solution.

### Experimental design

To examine the effect of DEX on HSR-induced lung injury and autophagy, the rats were divided into six groups (n = 5–12 per group). The sample size was determined by that used in a similar study^41^. In the Sham group (n = 12), incision and suture were performed on the left leg of the rat; only NS was used for tail vein injection, and no other drugs were used. The NS + HSR + DEX group (n = 12) was administered DEX at a dose of 5 µg/kg/h through the tail vein during the resuscitation stage of shed blood infusion, while the CQ + HSR + DEX group (n = 12) was administered 10 mg/kg of autophagy inhibitor CQ via an intraperitoneal injection 1 h before the operation, and the same DEX dose was also administered at the resuscitation stage. The NS + HSR + NS group (n = 12) was administered the same dose of NS instead of either CQ or DEX. The CQ + Sham group (n = 5) and CQ + HSR + NS group (n = 5) were respectively based on the Sham group and NS + HSR + NS group, using the same CQ injection method and dosage as the CQ + HSR + DEX group. At 24 h after resuscitation, the rats were euthanized via exsanguination (Fig. 1). To determine the lung wet/dry weight ratio, the left lung tissue was excised. The remaining lungs were immediately frozen using liquid nitrogen and stored at − 80 °C for further study.

### Histopathological examination

Twenty-four hours after the resuscitation of the HSR model, the upper lobe of the right lung was resected and fixed in 10% neutral buffered, embedded in paraffin, and then sectioned at 5-µm thickness. After deparaffinization and dehydration, the sections were stained with hematoxylin and eosin and examined under a microscope. According to the method used in a previous study^35^, 10 regions were randomly selected from each lung biopsy, and five uninformed auxiliary investigators were invited to score each region in terms of congestion, pulmonary edema, degree of cell infiltration, and degree of bleeding. The score was given on a four-point scale of 0 (none or normal), 1 (mild), 2 (moderate), or 3 (severe). The sum of the individual and lung injury scores of each aspect was calculated.

### Lung wet/dry weight ratio

Twenty-four hours after the resuscitation of the HSR model, the left lung tissue was weighed (wet weight) and then dried at 110 °C for 24 h. The dried tissue was weighed again (dry weight), and the lung wet/dry weight comparison acted as an indicator of pulmonary edema^39,42^.

### Protein extraction

Total proteins were isolated from part of the left lobe of the lung obtained 24 h after completion of the HSR model using T-PER (Tissue Protein Extraction Reagent) (Thermo Fisher SCIENTIFIC, USA) according to the manufacturer’s protocol. Briefly, lung tissue was homogenized with T-PER containing 5 mM dithiothreitol (DTT), 5 mM ethylene diamine tetra acetic acid (EDTA), and protease inhibitor (cOmplete; Roche Diagnostics GmbH, Sigma-Aldrich, IN, Germany) and centrifuged at 10,000×*g* at 4 °C for 30 min. The supernatant was collected and stored for subsequent analysis. Protein concentrations in lung homogenates were determined using a Pierce BCA™ Protein Assay Kit (Pierce, Rockford, IL, USA), according to the manufacturer’s instructions, using a multimode plate reader (Nivo 5 Multimode Microplate Reader, PerkinElmer, USA).

### Western blot analysis

Samples containing approximately 50 µg of protein were loaded onto 10, 12, or 15% sodium dodecyl sulfate (SDS)-polyacrylamide gels. After electrophoresis, the proteins were transferred to Amersham Hybond-polyvinylidene fluoride membranes (GE Healthcare Life Sciences, Germany), which were blocked at room temperature for 1 h with 4% (w/v) BlockAce (DS Pharma Biomedical Co., Ltd., Osaka, Japan). The membranes were then incubated with primary antibodies against ATG5 (mouse anti-ATG5 polyclonal antibody: sc-133158, Santa CRUZ, 1:1000 dilution), Beclin-1 (mouse anti-Beclin-1 polyclonal antibody: sc-48341, Santa CRUZ, 1:1000 dilution), P62 (rabbit anti-P62 polyclonal antibody: PM045, MBL, 1:1000 dilution), LC3 (rabbit anti-LC3 polyclonal antibody: PM036, MBL, 1:1000 dilution), and GAPDH (rabbit anti-GAPDH polyclonal antibody: sc-25778, SANTA CRUZ, 1:5000 dilution) at 4 °C for 12 h. After the membranes were washed with Tris-buffered saline with Tween 20, they were incubated with goat anti-mouse IgG-HRP (sc-2005; Santa Cruz, 1:10,000 dilution) or goat anti-rabbit IgG-HRP (ab6721; Abcam, 1:10,000 dilution) secondary antibodies at room temperature for 1 h. The membranes were then reacted with Clarity Western ECL Substrate (Bio-Rad, USA), according to the manufacturer's instructions. The membranes were scanned with an image scanner (ChemiDoc XRS Plus Imaging System, Bio-Rad). Exposure time was determined automatically. Densitometry was performed using analysis software (Image Lab Version 5.0, Bio-Rad).

### Statistical analysis

Data statistics and analyses were performed using GraphPad Prism 9 (GraphPad Software Inc., San Diego, CA, USA). Data are presented as mean ± standard error. Statistical analysis was performed using one-way ANOVA followed by the Tukey–Kramer multiple-comparisons test, as appropriate. Differences were considered statistically significant at *p* < 0.05.

## Data Availability

The datasets generated and analyzed during the current study are available from the corresponding author upon reasonable request.

## References

[1] Cannon JW (2018). Hemorrhagic shock. N. Engl. J. Med..

[2] Hooper N, Armstrong TJ, Hooper N, Armstrong TJ (2021). Hemorrhagic shock. StatPearls.

[3] Dewar D, Moore FA, Moore EE, Balogh Z (2009). Postinjury multiple organ failure. Injury.

[4] Ciesla DJ (2006). Decreased progression of postinjury lung dysfunction to the acute respiratory distress syndrome and multiple organ failure. Surgery.

[5] Ware LB (2006). Pathophysiology of acute lung injury and the acute respiratory distress syndrome. Semin. Respir. Crit. Care Med..

[6] Thompson BT, Chambers RC, Liu KD (2017). Acute respiratory distress syndrome. N. Engl. J. Med..

[7] Jarrar DORAID, Chaudry IH, Wang PING (1999). Organ dysfunction following hemorrhage and sepsis: Mechanisms and therapeutic approaches. Int. J. Mol. Med..

[8] Bhana N, Goa KL, McClellan KJ (2000). Dexmedetomidine. Drugs.

[9] Zhang Y (2017). Dexmedetomidine may upregulate the expression of caveolin-1 in lung tissues of rats with sepsis and improve the short-term outcome. Mol. Med. Rep..

[10] Shen J, Fu G, Jiang L, Xu J, Li L (2013). Effect of dexmedetomidine pretreatment on lung injury following intestinal ischemia-reperfusion. Exp. Ther. Med..

[11] Kılıç K (2012). The effects of dexmedetomidine on mesenteric arterial occlusion-associated gut ischemia and reperfusion-induced gut and kidney injury in rabbits. J. Surg. Res..

[12] Gu J (2011). Dexmedetomidine attenuates remote lung injury induced by renal ischemia-reperfusion in mice. Acta Anaesthesiol. Scand..

[13] Kobayashi A (2022). Dexmedetomidine suppresses serum syndecan-1 elevation and improves survival in a rat hemorrhagic shock model. Exp. Anim..

[14] Klionsky DJ (2005). The molecular machinery of autophagy: Unanswered questions. J. Cell Sci..

[15] Mizushima N (2007). Autophagy: Process and function. Genes Dev..

[16] Deretic V, Levine B (2018). Autophagy balances inflammation in innate immunity. Autophagy.

[17] Yu T (2019). Dexmedetomidine prevents septic myocardial dysfunction in rats via activation of α7nAChR and PI3K/Akt-mediated autophagy. Biochem. Pharmacol..

[18] Mauthe M (2018). Chloroquine inhibits autophagic flux by decreasing autophagosome-lysosome fusion. Autophagy.

[19] Yoshioka A (2008). LC3, an autophagosome marker, is highly expressed in gastrointestinal cancers. Int. J. Oncol..

[20] Pugsley HR (2017). Assessing autophagic flux by measuring LC3, p62, and LAMP1 co-localization using multispectral imaging flow cytometry. J. Vis. Exp..

[21] Wu S (2015). Expression and clinical significances of Beclin1, LC3 and mTOR in colorectal cancer. Int. J. Exp. Pathol..

[22] Ye X, Zhou XJ, Zhang H (2018). Exploring the role of autophagy-related gene 5 (ATG5) yields important insights into autophagy in autoimmune/autoinflammatory diseases. Front. Immunol..

[23] Peitzman AB (1995). Hemorrhagic shock. Curr. Probl. Surg..

[24] Fink MP (2002). Reactive oxygen species as mediators of organ dysfunction caused by sepsis, acute respiratory distress syndrome, or hemorrhagic shock: Potential benefits of resuscitation with Ringer's ethyl pyruvate solution. Curr. Opin. Clin. Nutr. Metab. Care.

[25] Laker RC (2017). Ampk phosphorylation of Ulk1 is required for targeting of mitochondria to lysosomes in exercise-induced mitophagy. Nat. Commun..

[26] Sun Y, Jiang C, Jiang J, Qiu L (2017). Dexmedetomidine protects mice against myocardium ischaemic/reperfusion injury by activating an AMPK/PI3K/Akt/eNOS pathway. Clin. Exp. Pharmacol. Physiol..

[27] Zhao Y (2020). Dexmedetomidine protects against lipopolysaccharide-induced acute kidney injury by enhancing autophagy through inhibition of the PI3K/AKT/mTOR pathway. Front. Pharmacol..

[28] Li ZB, Li GC, Qin J (2021). Dexmedetomidine attenuates lung injury in toxic shock rats by inhibiting inflammation and autophagy. Arch. Med. Res..

[29] Zhao S (2022). Protective effects of dexmedetomidine in vital organ injury: Crucial roles of autophagy. Cell. Mol. Biol. Lett..

[30] Zhang Y, Liu G, Dull RO, Schwartz DE, Hu G (2014). Autophagy in pulmonary macrophages mediates lung inflammatory injury via NLRP3 inflammasome activation during mechanical ventilation. Am. J. Physiol. Lung Cell Mol. Physiol..

[31] Lin L (2016). Time-dependent changes of autophagy and apoptosis in lipopolysaccharide-induced rat acute lung injury. Iran. J. Basic Med. Sci..

[32] Codogno P, Mehrpour M, Proikas-Cezanne T (2012). Canonical and non-canonical autophagy: Variations on a common theme of self-eating?. Nat. Rev. Mol. Cell Biol..

[33] Xiao Y (2021). Dexmedetomidine protects human cardiomyocytes against ischemia-reperfusion injury through α2-adrenergic receptor/AMPK-dependent autophagy. Front. Pharmacol..

[34] Ferguson ND (2012). The Berlin definition of ARDS: An expanded rationale, justification, and supplementary material. Intensive Care Med..

[35] Kumada Y (2019). Therapeutic effect of carbon monoxide-releasing molecule-3 on acute lung injury after hemorrhagic shock and resuscitation. Exp. Ther. Med..

[36] Matute-Bello G (2011). An official American Thoracic Society workshop report: Features and measurements of experimental acute lung injury in animals. Am. J. Respir. Cell Mol. Biol..

[37] Percie du Sert N (2020). The ARRIVE guidelines 2.0: Updated guidelines for reporting animal research. J. Cereb. Blood Flow Metab..

[38] Underwood W, Anthony R (2020). AVMA guidelines for the euthanasia of animals: 2020 edition. Retriev. March.

[39] Inoue K (2008). Protective role of heme oxygenase 1 in the intestinal tissue injury in hemorrhagic shock in rats. Shock.

[40] Maeshima K (2005). Prevention of hemorrhagic shock-induced lung injury by heme arginate treatment in rats. Biochem. Pharmacol..

[41] Jiang L, Li L, Shen J, Qi Z, Guo L (2014). Effect of dexmedetomidine on lung ischemia-reperfusion injury. Mol. Med. Rep..

[42] Kanagawa F (2010). Protective effect of carbon monoxide inhalation on lung injury after hemorrhagic shock/resuscitation in rats. J. Trauma Acute Care Surg..

